# Clinical Practice and the Attitude of Ophthalmologists Regarding Amblyopia in the Population of Jordan: A Cross-Sectional Study

**DOI:** 10.7759/cureus.30114

**Published:** 2022-10-09

**Authors:** Bahaa Al-din Jaber

**Affiliations:** 1 Optometry Science, Al-Ahliyya Amman University, Amman, JOR

**Keywords:** strabismus, ophthalmologists, knowledge, jordan, awareness, amblyopia

## Abstract

Aim

The study was aimed at finding out the knowledge and attitude among ophthalmologists regarding amblyopia in the population of Jordan.

Methodology

This was an observational cross-sectional study conducted in 2021 among ophthalmologists who live and work in the local society of Jordan and were aged between 30 and 50 years. The survey was conducted using an online questionnaire administered through email and various social media platforms. The level of knowledge and awareness of amblyopia, its causes, methods of treatment, and the best age for treating amblyopia was assessed.

Results

Out of 200 participants, the majority were in the age group of 30-34 years (47.5%). The male:female ratio was 1:1. The average better age for examination and treatment of amblyopia was three to five years (46%). The most common cause of amblyopia was strabismic amblyopia (50%). Children's and parents' cooperation most significantly influenced the treatment's success (49%). The preferred assessment was cycloplegic refraction (56%), and the preferred patching treatment was a direct cover placed over the eye (77%). The average time to treat amblyopia was less than three years in the majority (53.5%). The most critical factor determining the success of treatment was when the treatment began (57.5%).

Conclusions

Our study found that although the majority of the participants were less experienced overall, they had a good knowledge of the amblyopia age group and the method of examination. Still, there was a mixed response regarding the treatment. Hence there is a need to increase awareness regarding amblyopia by Jordanian ophthalmologists, social organizations, and educational institutes.

## Introduction

Amblyopia, also known as the lazy eye, is an evolutionary problem in the brain and not an organic neurological problem associated with the eye [1]. It is a visual deficit in which the brain fails to process the inputs [2]. Over time, the brain favors the dominant eye. It can be unilateral or bilateral, and there can be physical or pathological defects. This results in a decrease in visual acuity, which can go undetected [3]. The prevalence of amblyopia in the community ranges between 1-4%, and it is one of the most common ocular pathologies among children [4]. One of the recent studies documented the pooled prevalence of amblyopia to be 1.75% [5]. A meta-analysis of 60 children reported the prevalence of amblyopia to be 1.44% [6]. The reported prevalence in Europe is 2.9%, North America is 2.41%, Asia is 1.09%, and Africa is 0.72% [7]. Children between 0 and seven years of age are most susceptible. Amblyopia can be classified as strabismus, refractive, deprivation, or reverse [3].

The leading cause of visual impairment in children is amblyopia. It is one of the most common causes of unilateral partial visual acuity loss. Although many diagnostic modalities are available for amblyopia, it is still underreported [8]. If the diagnosis is delayed, it can lead to a decrease in contrast sensitivity and vision loss over time. This hampers the mental and social growth of the child, including skill development, school performance, confidence development, and career opportunities [9]. Varied treatment options for amblyopia management have been proposed. They can be alternate eye patching, glasses, contact lenses, cycloplegic therapy, dichoptic training, and penalization therapy [10]. Prompt diagnosis and timely treatment are essential for good patient outcomes in amblyopia. Lack of treatment compliance and follow-up usually results in vision impairment in these patients [11]. The majority of the previous studies have shown that parental knowledge is low regarding the management of amblyopia in children. In a study conducted to investigate the knowledge and attitudes regarding amblyopia among parents in Jeddah, Saudi Arabia, a knowledge gap about fundamental aspects of amblyopia was found and there was a need to strengthen the school screening programs [12]. It is imperative that ophthalmologists have adequate knowledge and awareness regarding amblyopia management to have a perfect outcome. There is an interest in research regarding the perceptions and clinical practice patterns of amblyopia among pediatric ophthalmologists [8]. To the best of our knowledge and as per the detailed literature review, there is no validated questionnaire regarding the assessment of knowledge and awareness regarding amblyopia among the health care ophthalmologists in the local community of Jordan. The study was aimed at assessing the perception, understanding, and awareness of ophthalmologists regarding amblyopia examination and treatment in children with the help of a predesigned questionnaire distributed online to the target population.

## Materials and methods

This was an observational, cross-sectional, questionnaire-based study conducted among ophthalmologists in Jordan regarding amblyopia knowledge and awareness during the year 2022. The institutional Ethics Committee approval was obtained from the institutional review board of Al-Ahliyya Amman University (approval number FAMS-OPT-2022). Appropriate informed consent was obtained from the participants before the study.

The study was performed using a structured questionnaire, which was subdivided into four parts: (1) Demographic data of the ophthalmologists in the form of age, gender, and work experience; (2) Knowledge-related questions to assess the ophthalmologists' knowledge, such as the average age in children to begin amblyopia examination and treatment; the tests that should be performed for the diagnosis of amblyopia in children; the most common cause of amblyopia in children in their locality; and the most common modality (coverage) of treatment for amblyopia in children; (3) The questionnaire also inquired about the extent of the effectiveness of the method of treatment followed in the amblyopic eye and the extent of cooperation by parents in applying instructions. The doctors were also asked to answer questions on treatment options for amblyopia after the age of 12 and about their knowledge of using digital glasses in the treatment of amblyopia. (4) There were a range of miscellaneous questions about the availability of early screening tests for amblyopia at home, an assessment of the extent of community awareness to deal with children's visual health, and a rating for this.

The authors framed the questionnaire with the help and consultation of two more ophthalmologists and validated the content. The questionnaire was disseminated to the target population through online platforms like email, WhatsApp, Facebook Messenger, and Telegram. Multiple emails were sent to ophthalmologists in the local community of Jordan to promote the survey and get the maximum number of responses. Only a single response was recorded from each ophthalmologist, and in the case of duplication, the extra answer was excluded.

We used IBM Statistical Package for the Social Sciences (SPSS) Statistics for Windows, version 21.0. Armonk, NY: IBM Corp. In our analysis, we used the mean (± standard deviation) to describe continuous variables such as age. We used the count (frequency) to describe other nominal variables (e.g., gender). We used the chi-squared test to analyze the difference in response between men and women. We used an independent sample t-test to determine the mean difference in age and knowledge of amblyopia, and we presented the data as a mean (standard deviation). All underlying assumptions were met unless otherwise indicated. We adopted a p-value of 0.05 as a significant threshold.

## Results

The questionnaires were sent to 450 ophthalmologists who practice in Jordan. A total of 200 responses were recorded (response rate = 44.4%). A total of 95 (47.5%) ophthalmologists were in the age group of 30-34 years, 75 (37.5%) in the 35-40-year age group, 24 (12%) in the 41-45-year age group, and 6 (3%) in the 46-50-year age group. With regards to professional experience, 86 had an experience of fewer than five years, 45 had an experience of five to 10 years, 42 had 11 to 12 years of experience, nine had an experience of over 20 years, and there were 18 ophthalmologists who had no professional experience after their graduation.

About 66 (33%) ophthalmologists believed that the average age to start amblyopia treatment in children was three to five years, 92 (46%) believed it was between six and eight years, 18 (9%) believed it was between nine and 11 years, and 24 (12%) thought it to be above 12 years. When asked about the critical investigations needed for amblyopia, 44 (22%) ophthalmologists said early refraction, 112 (56%) said cycloplegic refraction was the most important, 4 (2%) said the post-mydriatic test, 6 (3%) said the stereoscopic examination, and 24 (12%) said the visual acuity examination. A total of 30 (15%) ophthalmologists believed refractive error to be the most common cause of amblyopia, 38 (19%) believed it to be visual deprivation, 100 (50%) answered strabismus, and 32 (16%) believed errors in treatment as the leading cause for amblyopia. Considering the treatment of amblyopia, a large chunk of 144 (77%) ophthalmologists thought patching to be the best treatment for amblyopia based on their experience, eight (4%) believed contact lenses to be the best solution, 34 (17%) thought cycloplegic drugs, and four (2%) thought it to be other treatment modalities.

A total of 12 (6%) ophthalmologists thought the cause of amblyopia was the most important factor in determining the final treatment outcome; 48 (24%) thought the age when the treatment is started was the most important factor; 126 (63%) thought visual acuity was the most important factor; and the rest, 14 (7%), thought there were other factors. The respondents were also asked about the treatment duration of their amblyopic cases. The average duration of treatment for the cases that were dealt with was as follows: 107 said that it would require less than three years (53.5%), 53 said that it would require four years (26.5%), 21 said that it would take five years (10.5%), three said it would take six years (1.5%), and 16 said that it would take seven years (8%). The most significant factor in the treatment success of amblyopic cases was also investigated. A total of 115 (57.5%) answered that it was dependent on when treatment began, and 16 (8%) answered that it was based on the method of treatment. 55 (27.5%) answered that parent-child cooperation mattered, 12 (6%) said the cause of amblyopia could matter, and two (1%) said other reasons were the most significant factors in treatment success.

The proportional effectiveness of the treatment method used in the amblyopic eye was also investigated. The treatment was very effective as per 42 (21%), effective as per 130 (65%), and minimally effective as per 24 (12%). None said it was not effective, and four (2%) were not sure. The proportion of parental cooperation in applying instructions for the treatment of children with amblyopia was also investigated. It was excellent as per 20 (10%), very good as per 98 (49%), good as per 61 (30.5%), acceptable as per 16 (8%), and weak as per three (1.5%). The ophthalmologists were also asked if there is a treatment for amblyopia after the age of 12. A total of 82 (42%) said yes, and 118 (59%) said no.

On enquiring about using digital glasses in treatment for amblyopia, 167 (83.5%) said no, and 33 (16.5%) said yes. A total of 143 (71.5%) said yes to the idea that there is an early screening test to diagnose amblyopia at home, and 57 (28.5%) said no. The ophthalmologists were also asked about assessing the extent of community awareness regarding how to deal with children's visual health; the results are shown in Table 1.

**Table 1:** Community awareness level toward children's vision health according to ophthalmologists in Jordan (n=200).

Level of awareness	Response rate (%)
Excellent	4.5
Very good	23.5
Good	34.5
Acceptable	26
Weak	11.5

## Discussion

The study aimed to assess the knowledge and awareness regarding amblyopia among ophthalmologists in Jordan. Prompt diagnosis and meticulous management of amblyopia can have a better visual prognosis in the child's growing years [13]. A lack of knowledge and awareness among healthcare ophthalmologists can adversely affect the management outcome and have an adverse impact on amblyopic children needing attention [14].

The majority of the previous studies have surveyed parents and the general population. Amblyopia awareness was reported to be 10% among the general population and 50% among those attending pediatric and ophthalmology clinics in Saudi Arabia [15, 16]. However, we assessed the knowledge and awareness among the ophthalmologists, which makes this study different as it considers the point of view of the medical professionals, which would impact the assessment and management strategies adopted [15,16]. To the best of our knowledge, this is the first-ever study of amblyopia among ophthalmologists in Jordan.

In this study, we had 200 respondents of varied ages and experiences, indicating the diversity of ophthalmologists from various parts of Jordan. The knowledge and awareness level of our study population was average based on the response to the question, which is greater than that reported in India (3%) [17], Nigeria (2.9%) [18], and Saudi Arabia (10%) [15], but less as compared to the study published in Jeddah (50%) [16]. This suggests that ophthalmologists had good knowledge and were well-informed regarding amblyopia.

A good number of respondents, about 33%, knew that starting the amblyopia treatment at an early stage (three to five years) helped treat the condition. The majority (67%) believed that the average age for treatment was over five years, which is likely because a large number of ophthalmologists and postgraduate students responded to the questionnaire. In a study conducted in Northern Ireland, the average age for treatment was nine to 10 years old. Strabismic amblyopia was the most prevalent type of persistent amblyopia. Amblyopia was associated with socio-economic disadvantage and poor spectacle compliance and cooperation [1]. In studies from Europe and North America, parents' knowledge regarding amblyopia, squint, and patching treatment was found to be moderate [19]. Similarly, a good number of respondents knew that the primary investigation for amblyopia was cycloplegic refraction (66%), indicating that the majority were aware of amblyopia management tools. A total of 50% of respondents answered strabismus as the leading cause of amblyopia. Approximately 77% answered patching as the primary treatment, indicating a high knowledge level among ophthalmologists. Similarly, 63% believed that low visual acuity was the most common cause for determining the final visual outcome of amblyopia treatment. A total of 53.5% of respondents have dealt with that requirement for less than three years, probably due to increased compliance and the suitable modality of treatment.

A total of 57.5% believed that the most prominent factor in amblyopia treatment was when the treatment began because early diagnosis and prompt treatment can reverse amblyopia with excellent results. Approximately 65% of respondents believed that the treatment was effective, probably because of the good results and regular follow-up and compliance of the patients. About 80% of the ophthalmologists saw excellent compliance from parents regarding amblyopia treatment. This shows that parents were educated, understood the nature of the disease, and had compliance with treatment and follow-ups. Miscellaneous questions, like amblyopia treatment after 12 years, digital glass use for amblyopia treatment, and early screening tests, had mixed responses from the ophthalmologists, probably due to a lack of awareness regarding these questions.

The strengths of our study were its prospective nature and large sample size of ophthalmologists.

Limitations

The limitations of our study were that subjects were not recruited from all ophthalmology practices in Jordan. This study was critical to understanding the knowledge and awareness of the ophthalmologists in Jordan to get an idea of amblyopia treatment in the country. There is a shortage of good studies regarding amblyopia prevalence, management, and treatment in Jordan, and this forms the basis of future studies among the Jordanian population.

## Conclusions

Amblyopia is a severe ocular problem that needs to be addressed urgently. It can affect a child's vision, which can have irreversible sequelae. This study gave deeper insights into the knowledge and awareness of ophthalmologists regarding amblyopia. The knowledge and understanding of respondents were reasonably fair. In the future, more intricate measures will need to be taken to ensure that all ophthalmologists are well-versed in amblyopia diagnosis and management. Ophthalmologists need to be actively involved and parents to improve compliance and treatment outcomes. Regular screening can be performed, and teachers and school staff can participate in this campaign.
